# Measles, Media and Memory: Journalism’s Role in Framing Collective Memory of Disease

**DOI:** 10.1007/s10912-021-09705-2

**Published:** 2021-08-03

**Authors:** Elena Conis, Sarah Hoenicke

**Affiliations:** 1 UC Berkeley Graduate School of Journalism, Berkeley, CA USA; 2 UC Irvine, Department of Comparative Literature, Irvine, CA USA

**Keywords:** Journalism, Framing, Presentism, Memory, Disease, Measles

## Abstract

Language used to describe measles in the press has altered significantly over the last sixty years, a shift that reflects changing perceptions of the disease within the medical community as well as broader changes in public health discourse. California, one of the most populous U.S. states and seat of the 2015 measles outbreak originating at Disneyland, presents an opportunity for observing these changes. This article offers a longitudinal case study of five decades of measles news coverage by the *Los Angeles Times* and the *San Francisco Chronicle*, which represented two of the largest news markets in California when the measles vaccine was released, in 1963, and during the 2015 outbreak. Measles reporting during this period displays patterns pointing to an active role for journalists in shaping public understanding of health and medical matters, especially as they recede from public memory, through the employment of available and circulating political and cultural frames. Moreover, journalistic frames in this period of reporting incorporated presentist descriptions of the disease, which imposed present values on the medical past, and which were constructed of decontextualized historical references that supported prevailing contemporary notions of the disease. Framing and the tendency toward presentism, in the context of shifting public health discourse, had the effect of communicating an increasingly severe sounding disease over time, and of shifting blame for that disease’s spread from nature to government to individuals. Journalistic framing and causal stories have much power to shape public understanding of medical matters as they recede from public memory.

In 2015, a case of measles in a Disneyland visitor sparked an outbreak of one hundred forty-five cases in seven states. In the attendant media storm, Disney’s hometown paper, the *Los Angeles Times*, blamed the outbreak on parents. Measles was a leading, highly contagious childhood disease that once killed millions each year, wrote *Times* reporter Karen Kaplan. But modern Americans no longer feared it because they “have no first-hand experience with measles or other dangerous diseases that used to be common.” The problem, Kaplan explained, was exacerbated by the influence “of the anti-vaccination movement,” which spread misinformation about measles and its vaccine and was “certainly” responsible for the outbreak’s “scope” (Kaplan 2015). Like others published at the time, the article defined measles as a social problem and identified its cause as misinformation spread by individuals. The article also implied journalism’s role in the solution: to reach a wide audience with reliable facts about measles in the present as well as pertinent information about measles’ past.

As a disease that has been vaccine-preventable since the 1960s, measles has been the subject of intermittent periods of intensified media coverage for more than a half-century, with coverage usually triggered by outbreaks or vaccine issues. This essay offers a longitudinal case study of five decades of measles news coverage by two California news outlets, the *Los Angeles Times* and the *San Francisco Chronicle*, which represented two of the largest news markets in the state at the time the measles vaccine was released, in 1963, and at the time of the Disneyland outbreak in 2015.^1^ Throughout this period, measles reporting displays patterns that point to an active role for journalists in shaping public understanding of health and medical matters through the employment of available and circulating political and cultural frames.

Journalists also played a role in shaping public understanding of measles’ past. Because measles was a disease in decline, it was increasingly familiar through collective, not autobiographical, memory. Its collective memory was forged, in large part, through journalistic frames that lent themselves to presentist descriptions of measles’ history. Often, this history was constructed out of fragmented and decontextualized references, selected because they reflected contemporary values and supported contemporary frames. The act of framing and the tendency toward presentism had the effect, over time, of shifting responsibility for the disease’s spread from nature to government to individuals. Moreover, as this shift took place, journalists described measles as more severe than their predecessors had. As agency shifted, therefore, the individual became responsible for the spread of a more serious-sounding disease than nature had been responsible for in the pre-vaccine era. This longitudinal case study supports findings other scholars have produced regarding the episodic, decontextualized and presentist nature of reporting on social problems over time, as it demonstrates a significant role for journalists in shaping public understanding of social problems in the realm of health and medicine.

## Frames and blame

Deborah Stone’s typology of causal stories offers a useful starting point for analyzing journalistic coverage of measles as a social problem. Per Stone, narratives about political problems highlight either accidental, inadvertent, mechanical, or intentional causes (1989). In the earliest post-vaccine phase of reporting, measles was, temporarily, an accident of nature, newly avoidable via vaccination. In the subsequent reporting phase, however, journalists offered causal stories that pointed to inadvertent human causes of measles’ persistence: individuals who did not know or understand the value of vaccination, or political agents who did not realize that decisions to cut budgets or programs would spread disease. In the final phase, journalists circulated a story that spoke to intentional cause: individuals who deliberately spread or believed misinformation about measles and vaccines. For Stone, the inadvertent cause is a common liberal explanation of problems and the intentional cause a conservative one, but these causal stories appear in both the *Times* and the *Chronicle*, despite the papers’ divergent politics. Moreover, as the media’s causal stories skewed conservative over time, vaccination policy skewed increasingly liberal through programs that expanded federal involvement in oversight and provision and through state policies that expanded local authority to require vaccination (Colgrove 2006; Conis 2015).

Causal stories, which are often handed to journalists by policy actors, officials, and experts, are often shaped through framing. In mass communications theory, frames emphasize a particular interpretation of a problem, its causes, and its possible solutions. Journalists, frequently unaware of or insensitive to the presence of framing, may inadvertently communicate news in ways that favor a particular frame. Consequently, argues Robert Entman, “the frame in a news text is really the imprint of power”—the power of the party served by the frame (1993, 55). If framing provides an explanation of the forces and factors giving salience to issues, then journalists, by communicating and circulating frames, play a key role in defining the importance of social issues, their causes, and the promise of their various solutions.

Mass communication theory’s definition of framing is related to but distinct from framing as understood by the discipline of history and specifically the subfield of medical history. Both theories posit framing as a phenomenon that advances problem definitions and causal solutions. Focused less exclusively on textual communication, however, framing in medical history describes a multi-sited series of events that shape human understanding of a health or medical matter, its causes, and its optimal management at a particular moment in time. Biology, language, identity, politics, economics, and contemporary scientific knowledge, for example, cofunction to frame a biological situation as a “disease” or condition in need of medical or scientific attention (Rosenberg 1992). Failure to perceive the frame, according to Charles Rosenberg, risks presentism, the imposition of present values and knowledge on the interpretation of medical situations past.

The analysis in this essay draws on both theoretical frameworks to examine the framing of measles in its post-vaccine era in the single site of the news media, focusing on the form and function of those frames over time. Our analysis finds that over five decades of measles coverage, the frames for measles reporting continually shifted, as briefly noted above and described in detail below. As the frames in measles reporting shifted, the reporting itself was characterized by presentist representations of measles’ past, which supported the frames journalists used to make sense of measles in their own time. For journalists, measles’ history—the number of cases the disease formerly caused, its victims, its symptoms, and its complications—helped define measles as a contemporary problem and called up prevention as an imperative present solution.

Measles’ history was an important reference point partly because measles incidence had significantly declined in the decades *before* the vaccine was introduced. But because of this decline, journalists offered historical facts about measles excised, for example, from the disease’s own recent history of treatment and management. Journalists’ construction of measles’ history from decontextualized fragments echoes a pattern that other scholars have documented regarding media coverage of historical events, applied here to a topic—infectious disease management—sometimes viewed as less politicized than others (Zelizer 1998; Edy 2006). It also echoes other patterns from social issue reporting. As the past becomes more distant, Jill Edy has argued, it may become increasingly subject to media manipulation; at the same time, the collective memory journalists help construct, itself a product of framing, can become a frame on that past (2006, 16). Vaccination-era measles reporting shows both patterns holding.

Reporters who covered measles in the vaccine era constructed its past as increasingly troubled over time. Their reporting reveals a pattern of referencing increasingly larger numbers of measles cases, as well as deaths, attributable to the disease historically. They also described the disease’s symptoms and consequences in increasingly severe terms; while some of the earliest vaccination-era stories referred to measles’ reputation for being “benign,” later reports would refer to it as deadly and fatal, never mild (Nelson 1964a). As reporters constructed this increasingly collective memory of measles to replace its receding autobiographical memory, the memory itself, of a severe and deadly disease, became a resource they or other journalists used to explain the current measles-related events they were covering (Halbwachs 1992; Edy 2006; Lang and Lang 1989).

Journalists, adds Edy, tend to present the past as fact carrying the “mantle of objectivity,” as opposed to selective and embedded truths open to interpretation (2006: 164). In medical or scientific reporting more generally, this mantle of objectivity may seem even more impenetrable than it might in other forms of social problem reporting (Li et al. 2018; Scheufele and Krause 2019). The number of measles cases, or deaths, in a particular year, for instance, reads as objective fact, but the embedded truth of that number—who was at highest risk of death, why, and how the number of deaths related to deaths from other causes, or deaths due to the same cause the previous year or decade—could greatly influence the meaning and implications the number carried and the causal stories it supported. In media reporting on measles, a collective memory of measles as severe and deadly quickly became a commonly deployed frame for journalistic stories whose message or “moral” concerned the urgency of measles prevention through vaccination (White 1987). Measles’ increasing severity also offers an example of how journalistic coverage of the past over time can “keep raising the threshold at which we begin to respond” (Zelizer 1998: 204).

From the 1960s through the 2010s, journalists deployed a series of evolving frames to explain measles’ persistence. Many suited presentist ends, as noted above; all also resonated with contemporaneously circulating political and cultural stories. A public ignorance frame in 1960s measles reporting spoke to a tension over the balance between physician and lay authority over individual and family health (Starr 1982; Tomes 2016). The urban poverty frame journalists used in the 1970s reflected President Lyndon Johnson’s Great Society ideals (Colgrove 2006; Blumenthal and Morone 2010). The frame of national and especially urban decline in 1990s reporting supported the liberal policy goals of President Bill Clinton’s administration, especially with respect to health care (Skocpol 1996; Starr 2011). The frames of elite privilege and the science wars in 2010s reporting echoed rhetorics then gaining currency in American politics and culture (Goldenberg 2016; Kazin 2017). Per Rosenberg, disease has always served as “an occasion and agenda for…debate about the relation among state policy, medical responsibility, and individual culpability” (1989). Journalistic measles coverage shows how journalists, wittingly or not, weighed in on such debates with reporting that framed measles through the prisms of prevailing policy and cultural discourse of the day.

The sections below analyze the frames and presentist themes apparent in three periods of measles reporting: the period of the measles vaccine’s introduction, 1963–1967; the period characterized by two attempts to eliminate measles from the U.S. between 1967 and 2000; and the post-elimination period, starting in 2000, when health officials declared measles no longer endemic to the U.S. We chose two newspapers that, in addition to serving large audiences per circulation numbers published in the *Ayer Directory of Publications* and *Gale Directory of Publications and Broadcast Media*, were consistently archived and catalogued and provided regular coverage over the entire period in question. To narrow our large dataset (more than four thousand articles mentioning measles across the two papers), we browsed state medical journals to identify years in which medical and health experts discussed significant developments in the disease’s prevalence or management; we then searched for measles news articles from these years, yielding a subset of three hundred nine articles. We used inductive and abductive approaches as well as thematic analysis to identify common threads across the data, such as references to measles’ past and reasons for non-vaccination in the present (Bowen 2006; Bryant and Charmaz 2007; Charmaz 2003).

## A new vaccine

Measles was about to make its “Last Stand,” the *San Francisco Chronicle*’s cover declared on March 19, 1963, just a day after the U.S. government announced the imminent licensure of several new measles vaccines (*San Francisco Chronicle* 1963). In the following days, journalists reported the news with a set of frames that highlighted the bountiful, exciting and superior nature of American science on the one hand and corporate largesse and government cooperation on the other. The *Los Angeles Times* reported that the “for real” medical drama of the vaccines’ development was going to be featured in a CBS special (MacMinn 1963). The *Chronicle* article noted that three American companies were behind the advance and that the government was taking the unusual step of eliminating a waiting period before the vaccines could come to market (*San Francisco Chronicle* 1963). The reason: measles sickened an estimated four million Americans each year, causing four hundred deaths annually, making it responsible for more deaths than polio, the most dreaded childhood disease of the day (*San Francisco Chronicle* 1963; Oshinsky 2005).

This framing—of American science as triumphant over a high-stakes medical problem—was familiar, as it had been used to report on the disease in the decades leading up to the vaccine. “Children will not die any more of measles,” the papers had declared in 1939 on the discovery of an antibiotic effective against pneumonia, a sometimes-fatal infection that struck children with measles (Associated Press 1939). Journalists covered the 1940s discovery of gamma globulin, an antibody-rich extraction of blood plasma treatment, with headlines that declared the “end” to “cycles of measles” (United Press 1944a, 1944b). By the early 1950s, the *Times* reported, the numbers of children dying annually from diseases such as measles, diphtheria and scarlet fever were down to just a handful from the “hundreds” of such deaths that had taken place in the 1920s, thanks to “medical strides” and “progress” (*Los Angeles Times* 1961). By the late 1950s, wrote the *Chronicle*’s syndicated physician-columnist, Walter Alvarez, the key problems were car accidents and poisonings, which killed “four times as many children under 4 years of age as formerly were killed by all the common infectious diseases put together!” (Alvarez 1958).

While the framing of measles treatment remained constant from pre- to post-vaccine reporting, the framing of the disease itself changed. Specifically, the story of measles as a vanquished foe gave way to the story of measles as a severe and ubiquitous killer, its number of cases, deaths, and associated complications all higher and more severe than previously described. Before the vaccine, the *Times* reported that measles killed from 385 to 522 Americans per year; after, the paper reported that measles killed “500 U.S. children every year and permanently injures many more” (Goddard 1963; *Los Angeles Times* 1963). Weeks before the vaccine was released, the *Chronicle* had reported “upwards of 400,000 cases” of measles annually, mentioning no deaths; after the vaccines’ release it reported four million annual cases and four hundred deaths, not specifying if these were domestic or global figures (Associated Press 1963a; Associated Press 1963b). Measles, the *Times* reported on the occasion of the vaccines’ licensure, killed twenty-five percent of its victims (*The Washington Post* 1963).

Both papers also began to include details about measles complications in their reporting. Of the reported four million cases, one in one thousand would suffer “damaged brains,” and some of those would be “severely mentally retarded,” wrote *Times* science reporter Harry Nelson (Nelson 1964b, 1965). Others reported that measles would leave many children deaf (Hersh 1963). By the time the fifth measles vaccine made it to market, in 1965, the *Times* reported that twenty million children were at risk of the disease and its life-altering and deadly complications and that the disease was now the “the No. 1 killer among childhood diseases” (*Los Angeles Times* 1965; Nelson 1965). This framing matched the scale of the threat to the power of the current technology. The vaccine was a far more effective scientific tool than early antibiotics and gamma globulin had been. Journalistic reports that amplified the numbers of measles cases, deaths, and complications refashioned the disease into a foe worth the vaccine’s unprecedented capacity for technological disease control.

As an event or reality recedes into the past, some members of any news audience will retain their own memories of it, notes Edy (2006: 13). Some journalists covering measles immediately post-vaccine therefore acknowledged—and explained—the new framing of measles in their reporting by setting the news of measles’ severity into larger frames of scientific advancement and physician authority. People tend to think of measles as “uncomfortable but harmless,” the *Chronicle*’s front-page noted (*San Francisco Chronicle* 1963). “Most parents and many doctors consider measles to be no more serious than a cold,” Nelson wrote in the *Times* (Nelson 1964b). Alvarez imagined “mothers” thinking, “why bother about measles? It isn’t much of a disease, and every child has to have it” (1965). But it was time to rethink measles, he urged, presenting the “surprising” information that in 1960 only sixty American children died of polio while four hundred eight died of measles. And “I was just reading,” he added, “that 50% of all children who have measles show some involvement of the brain” (1963). The following year, both papers carried the news that measles causes “mental retardation” for many (Alvarez 1965; Nelson 1964b). American scientific and medical experts, such stories communicated, were turning up new information about problems and solutions all the time; a parent’s (particularly mother’s) duty was to trust that expertise without question.

Framing involves selection and omission, and in framing measles as severe, journalists did both—wittingly or not. Stories offered selective information about measles’ toll in poor, foreign, and historic settings. Alvarez wrote of how the disease had killed thousands of “American Indians” and “grown natives” in the South Seas “in the old days” (Alvarez 1963). A *Times* story dubbed measles the “No. 1 killer,” neglecting to mention that this was true in foreign countries, not in the U.S. (Nelson 1964a). Other stories in the paper reported on deadly epidemics in India, Iran, and “some African countries,” where measles was responsible for a quarter of all deaths (United Press International 1964; Nelson 1964b, 1965). Such news, in addition to supporting narratives of measles as severe, supported the framing of the measles vaccine as an American scientific success story. In African countries such as Upper Volta, where the measles vaccine had been tested years before, the *Times* reported that the “U.S. vaccine” was now saving the lives of hundreds of children (Toth 1964). The history that Alvarez shared, meanwhile, turned on a “great American pathologist’s” discovery of the culprit virus (Alvarez 1963). This process of selection, again witting or not, allowed the dual frames of severe measles and American scientific superiority to support one another.

Measles began this period seen as an act of nature. But human action in the form of vaccination began to reconfigure measles into a social problem with a specific solution. That problem was defined through reference to poor, foreign, and historic contexts, and the frame of American scientific triumph pointed to the solution: wide use of the American vaccine. Narratives in this first phase of reporting thus upheld a very particular relationship between the present and the past: one in which the past showed the scale of the threat posed by nature, a threat matched only by the power of contemporary American technological capacity.

## Eradicating measles: a tale of two attempts

The second phase of reporting on measles—1967 to 2000—was characterized by repeated efforts to widely vaccinate U.S. children against the disease through two national eradication campaigns. During this period, the causal story in news reports shifted decidedly away from nature; the problem of measles’ spread now had inadvertent causes, including an innocently ignorant public and irresponsible government. These causes were buttressed by a strengthened severity frame, reinforced by the frames of American scientific superiority, middle class values, and government responsibility.

In 1967, President Lyndon Johnson announced that the Surgeon General would vaccinate up to ten million children and four million infants in order to eradicate measles by 1968 (San Francisco Examiner 1967; *San Francisco Chronicle* 1967). Journalists—now describing an infection that “often has serious long term effects,” including pneumonia, deafness, encephalitis, “mental retardation and death”—reported that measles would very soon “join the ranks of the conquered diseases of childhood” (Sell 1967). Headlines called measles “a crippler” (*San Francisco Examiner and Chronicle* 1967). Reporters broke down the four million cases into jarring statistics: one in six would have “serious complications,” five hundred would die of encephalitis and pneumonia, and sixteen hundred would become “mentally retarded” (*San Francisco Examiner and Chronicle* 1967).

In the papers, the cause behind the shocking figures was not just measles itself but the parent who was unaware of measles’ severity, whose blameless ignorance posed a threat to middle-class stability and American technological achievement. It was true, said a physician in the *Chronicle*, that just a few years ago doctors told parents to let their children get the measles because they had to have it sometime and because it was better to “get it over with” while young. But with a measles vaccine available, “such advice is completely wrong,” he said (*San Francisco Examiner﻿* 1967). Cases fell significantly during the first few years after the eradication goal was declared, but then they began to climb again in the early 1970s. As they did, news reports described parents who didn’t vaccinate their children as “confused” about measles’ true dangers (*San Francisco Examiner* 1967; Associated Press 1971). Journalists offered stories from the burgeoning middle class that proved measles’ threat. “Pretty” hazel-eyed Kim Fisher from Fort Wayne, Indiana, had become a burden on her hardworking parents since measles encephalitis had left her “handicapped” (*San Francisco Examiner and Chronicle* 1967). Scotty Whitmire from Sacramento had to relinquish his dream of becoming the “world’s fastest runner” because a “rare measles virus” had left him unable to sit upright or speak (*Los Angeles Times* 1971). Fair-haired Paul House, pictured on his rocking horse on the *Chronicle*’s front page, threatened to delay Apollo 13’s launch when a backup pilot and astronaut were exposed to his measles (United Press and Associated Press 1970).

In 1976 and 1977, measles cases reached epidemic heights, and the causal story of the naively ignorant parent was supplanted by a causal story pointing to ineffective government. A state vaccination law was not working because officials were not enforcing it (Birkinshaw 1978). When a revision gave the law more teeth, the papers reported that with limited resources, schools and counties could only do so much (Nelson and Kistler 1977). California’s Proposition 13, said a school health director in the *Times*, had forced the district to cut the nurses who had vaccinated eighty thousand children the previous year; those shots would now “have to be obtained through a county clinic or a private doctor” (Thompson 1978). But that causal story faded when President Jimmy Carter announced a new eradication campaign, late in 1978. The allotted $45 million in federal funds, California reporters explained, would help states detect outbreaks and “crack down” on unimmunized children (Associated Press 1978).

As it had in the wake of the previous eradication campaign, measles fell and then climbed again in the 1980s. As it did so, two new causal stories emerged in quick succession. The first placed blame on nature within a frame of evolving scientific knowledge. Journalists explained “continuing outbreaks” by covering weakly protective older vaccines, poorly timed vaccines, vaccine failure, and measles’ tenacity. The measles virus outwitted vaccines six to forty percent of the time, reporters noted (Gaw 1989; *San Francisco Chronicle* 1989a). Scientists were “surprised” to learn that measles was more contagious and spread more easily through the air than previously thought; some children had become infected in doctors’ offices more than an hour after a measles patient had left (*San Francisco Chronicle* 1989b). Measles was proving “so transmissible that even groups with complete immunization circumstances may result in outbreak,” said a health official in the *Chronicle* (*San Francisco Chronicle* 1989e). This new scientific knowledge strengthened, once again, the disease severity frame.

The second causal story to emerge placed blame on government neglect of urban and particularly poor people of color and immigrant communities. Measles, the *Times* reported, was breaking out in “inner-city areas,” and the city’s cases were not evenly distributed: almost eighty percent struck “Latinos” (Steinbrook 1989; *Los Angeles Times* 1989). A front-page story told of “poor ethnic minorities” without access to medical care who feared American institutions and the discovery of their immigration status (Spiegel 1989). When measles struck the Samoan community in Los Angeles, the *Times* described a group “hit disproportionately with poverty, high unemployment and school dropout rates and youth crime” (Ford 1989). “These populations” needed higher immunization levels, said a health worker in the *Chronicle* (*San Francisco Chronicle* 1989d). Health officials had “been able to vaccinate only about 50 percent of children in poor urban areas,” the paper reported (*San Francisco Chronicle* 1989b). The problem was a statewide “money crunch,” explained the *Times*, caused by budget cuts and rising vaccine prices. Public clinics with short hours, long lines, and too little vaccine were “turning [children] away” (Gaw 1989; *San Francisco Chronicle* 1989c). The situation was so “desperate” that California was borrowing vaccines from neighboring states (Gaw 1989; *San Francisco Chronicle* 1989e).

This causal story called, as it had a decade before, for more and bigger government. Reporters covered a new policy approach to vaccinating children, a “universal” plan in which the federal government would assume the cost of vaccinating every child. “The nation that sold Coca-Cola to the world…can figure out a way to sell universal immunization,” said a doctor interviewed in the *Times* about a plan to make government the sole purchaser and provider of all vaccines (Fuetsch 1993). Supporting this notion (if unconsciously), journalists again invoked foreign, poverty-stricken contexts. This time, these contextual stories were brought in not to show how bad measles was in the past but to show how bad it had become in the modern U.S. “Measles has permanently established itself here in much the same way that it has in developing countries,” a *Times* writer wrote (Spiegel 1989). “It’s the kind of measles you see in Third World countries,” said a state health director in the paper (Spiegel 1989).

References to the past remained an aspect of measles reporting in this period, but the relevant past changed. The past still proved measles’ severity: as the *Times* noted, “as many as 500,000 children in the United States caught measles each year” before the 1960s (Spiegel 1989). (This number was, notably, not the four million cases cited in the first phase of reporting.) But the past was also now a reminder of how effectively measles had previously been controlled. California had enjoyed its “all-time low” of one hundred seventy-six measles cases in 1983 (Gaw 1989). Los Angeles hit a low of forty cases in 1986 (Spiegel 1989). As Edy notes, journalists engage in the process of creating pasts that “also become the resources we use to make sense of current events” (2006: 16). Presentism, in this case, shifted the moral of measles reporting. The moral of earlier reporting was that measles was too serious not to vaccinate against; by the late 1980s, the moral was that it was important to remember that this serious disease was controllable via widely accessible vaccination, which had been achieved in the past.

In 1993, President Bill Clinton signed a budget creating a partially universal vaccination program, which guaranteed childhood vaccines to low-income and uninsured children. Subsequently, the framing of measles’ persistence shifted again. If nature was the responsible agent in the first phase of reporting and government the primary responsible agent in the second, the third phase saw the culpable individual emerge, as a personal responsibility frame came to dominate. A 1996 *Chronicle* editorial, for example, pointed back to California’s fifty child measles deaths during the 1989 epidemics as a reminder that measles was deadly. But that was not the editorial’s main message. The deaths were “abhorrent” and a “stern reminder” that it was everyone’s business to make sure every young child was vaccinated, especially now that “vaccines are never denied to people who lack funds” (*San Francisco Chronicle* 1996). With the entitlement in place, anyone who failed to make the necessary “trip to a clinic or physicians’ office for a swift immunization” was personally responsible for any cases of disease that ensued (*San Francisco Chronicle* 1996).

## The era of elimination

The final phase of reporting was characterized by a pronounced narrative shift with what would come to be known as the “Disneyland outbreak.” The phase’s commencement was marked by measles’ elimination, declared in 2000, a time when the accidental story reigned. When measles returned in the form of a multi-state outbreak originating in California in late 2014 (which was when the Disneyland outbreak began), that accidental causal story was quickly replaced by an intentional one. As this shift took place, the disease severity frame persisted, upheld once again by presentist representations of measles’ past.

In 1999, the U.S. saw an all-time low of one hundred measles cases; California had just nine (Cimons 1999). Reporters shared the “news,” for the third time, that measles was poised to join “the ranks of…diseases that have been effectively wiped out in the United States” (Bynum 1999). By the following year, 2000, the few measles cases in the country were all imported from abroad, a fact that led federal health officials to declare the disease eliminated from the U.S. Measles, occurring in isolated instances in this period, was framed as foreign and historic, temporarily assigned, once again, to a causal story that placed blame on nature. Journalists reminded the public that measles “still kills a million children a year” in the “developing world,” and that the 1989–1991 outbreaks, when “Los Angeles was hit particularly hard,” proved measles’ might (Allen 2000). A local pediatrician told *Times* readers that “long ago, many families would have 10 to 15 children to have only a few survive” (Qaqundah 2000). Health officials, reporters noted, had to remain vigilant to ensure that “foreign” measles didn’t gain a “domestic toehold” (Allen 2000; Rosenblatt 2000).^2^

But measles’ causal stories shifted back to the realm of human agency during the 2015 Disneyland outbreak. The causal stories in news reporting on the outbreak emphasized the intentional acts of individuals and were supported by the persistent severity frame. At the same time, the set of collective memories about measles—constructed with selective fragments of information about measles’ pre-vaccine past and its post-vaccine outbreaks and control—now served as frames for stories addressing both measles’ severity and the uniquely egregious nature of the current outbreak.

Like Kaplan, journalists reporting on the Disneyland outbreak invoked measles’ pre-vaccine days to prove measles’ contagion and deadly capacity and to offer commentary on the present. In pre-vaccine days, journalists reported, measles infected forty thousand Californians a year, killing more than 2.6 million—worldwide (Allday 2015b; Kaplan 2015). Even in post-vaccine outbreaks, like those of 1989–1991, measles “ravaged California” (Lin II 2015). Journalistic references to the past in this period carried clear moral implications. The story of fifty thousand students suspended for lacking vaccines in 1977, who were back in school, vaccinated, “within days,” spoke to the sufficiently measles-fearful obedience of parents past, who quickly immunized their children once apprised of the disease threat (Lin II and Xia 2015). The 1989–1991 outbreaks were chalked up to “lax vaccination policies,” which, when strengthened, drove measles cases firmly down; this spoke to a call for stricter vaccination policies in the present—even as it omitted the circulating explanation of the invoked past, namely health services beyond reach of the poor and undocumented (Allday 2015a).

As in the earliest phase of measles reporting, numbers, especially historical figures, proved fungible, adapted to suit the persistent severity frame. A *Times* story reported that measles caused one death for every five hundred cases, and two cases of encephalitis for every five hundred cases, “many of those” resulting in “permanent brain damage” (Lin II 2015). (These were, notably, higher rates than those in earlier reporting, mentioned above.) Other journalists reported a figure of one or two deaths per thousand, not five hundred; but even in these cases, the reporters failed to specify that the figure represented measles’ death rate globally, where it was most prevalent, and fatal, in countries with poor health outcomes overall. The *Times* conflated the nation’s one hundred fifty thousand cases in the 1989–1991 outbreaks with those in California alone (Lin II 2015). The *Chronicle*, by contrast, reported that measles had spread from Disneyland before, causing a fourteen-person outbreak in 1982 and a five-person outbreak in 2001, numbers juxtaposed with the one hundred thirty-two cases connected to Disneyland by March 2015. Such past outbreaks were described as “small and self-contained” in comparison to the present one (Allday 2015a). But this representation of the past was made possible only by omitting the outbreaks of 1989–1991. The framing of measles’ historic severity and control, even if inconsistent across the two papers, consistently points to the media’s growing ability to manipulate the past as that past became increasingly remote.

In a new pattern in this period, however, reporters invoked the histories of other epidemic infectious diseases in order to illustrate measles’ severity through metaphor, and in doing so, they offered a causal story that placed epidemic blame on the negligent individual (Lupton 1995). Reporters described measles as more contagious than SARS and Ebola, likened it to smallpox, and in calling the index case “Patient Zero,” conjured up the early days of AIDS (Lin II 2015; Fagan 2015; McKay 2014). As in that epidemic, a reckless individual was held culpable (Allday 2015a). Though numerous reporters said health officials would not identify the person who sparked the outbreak, a *Times* editorial described “her” as an unvaccinated person with a highly contagious disease who traveled in the “tight confines of two airliners” before visiting “a tourist destination that draws people from throughout the world,” where she was “packed in with others at the theme park, including babies too young to have been vaccinated.” The editorial offered no explanation for her lack of vaccines but offered a causal story that pointed to the anti-vaccine movement with its “ignorant and self-absorbed rejection of science” and “anti-science stubbornness” (*Los Angeles Times* 2015).

Coverage of the outbreak reveals a common synecdoche for the movement, a negligent individual consistently described as a privileged, white, anti-science parent, usually female, who did not vaccinate her children (Conis 2015; Reich 2016; Hausman 2019; Goldenberg 2021). Both the *Times* and the *Chronicle* included, in their mix of outbreak coverage, stories on a new study that found the highest rates of under-immunization in *two* types of northern California communities: those with more graduate degrees and those with incomes below the poverty line (Brown 2015; Colliver 2015). But in outbreak reporting itself, the low-income individual was omitted as reporters focused on “upscale” and “affluent and coastal” communities where parents believed in “myths” and succumbed to “hysteria” (Allday and Colliver 2015; Foxhall 2015; Karlamangla 2015; Lin II and Xia 2015). The *Chronicle* reported that non-vaccinating individuals “clustered” in communities with “tony private schools”; their decision not to vaccinate was positioned as a luxury born of privilege (*San Francisco Chronicle* 2015a). The *Times* portrayed these parents as a threat to others: “I feel surrounded by women here in Santa Monica, where I live, who have chosen to not vaccinate their children for personal reasons,” said a quoted parent, “and…put their children at risk to infect others” (Xia et al 2015). “Score one for science,” read a *Chronicle* editorial later, after a state Senate panel voted to tighten California’s school vaccination law in response to the outbreak (*San Francisco Chronicle* 2015b).

## Conclusion

The severity frame in measles reporting persisted for decades partly because of measles’ own fade into memory and partly because it was complementary to other frames and adaptable to multiple causal stories. In the sixties, measles’ severity frame spoke to the need to place trust in experts and American scientific achievement; in the seventies, it spoke to a call for social uplift; in the late eighties and nineties, it supported the cause of greater government involvement in health care; in the 2010s, it resonated with frames of elite privilege of anti-science citizens. As measles’ framing shifted consistently in one direction, its causal story moved in and out of the realm of nature, from there to realms of human agency during outbreaks and back again when outbreaks or prevalence declined.

Stone (1989) suggests no teleology among causal stories, but a number of scholars have demonstrated the emergence of a neoliberal ethic, incorporating an emphasis on personal responsibility, in public health discourse, including the narratives employed to describe public health problems and their underlying causes (Lupton 1995; Galvin 2002; Ayo 2012). By the time of the Disneyland outbreak, personal responsibility (and also “investment”) in health was widely seen, as Nike Ayo notes, “as a willful obedience to the duties and obligations imposed by citizenship” (101). The “good” health citizen, adds Rose Galvin, is responsible, makes rational decisions, and participates in social life (2002). By placing blame on the irresponsible, anti-science individual, the emergence of a causal story identifying intentional blame for the 2015 measles outbreak fits into a broader shift in public health discourse across modes of communication. Journalists who reported on the outbreak by framing the story as one about non-vaccinating individuals deliberately rejecting science framed them as disobedient citizens. These were individuals who deserved sanction, not largesse. Indeed, what followed was a modification to California vaccination law that removed a clause granting a right to object to vaccination on the basis of personal belief.

When Governor Jerry Brown signed that bill, the move gave California one of the most stringent school vaccination laws in the nation. Vaccination rates increased; measles outbreaks stayed at bay; and measles receded from journalistic attention. But five years later, in 2019, U.S. measles cases exceeded levels not seen since prior to elimination (Ingraham 2019). California was spared more than a few isolated cases (a fact attributed to the 2015 law), but the nationwide cases still made local news, as did a cultural dispute over measles’ severity. A different California institution—Hollywood—featured in media coverage of this dispute. The *Chronicle* featured a story about a former sitcom actress reportedly “furious” that a show she once starred in, “The Brady Bunch,” was being shared on social media as proof that measles was once not considered serious. “If you have to get sick, sure can’t beat the measles,” the actress’s character said in a 1969 episode in which the Brady children were delighted that measles kept them home from school. As the meme spread online, the actress told reporters she was “really concerned” about the show’s transformation into a pro-measles meme and that “as a mother” she had vaccinated her own daughter (Sippell 2019).

Reporters following the story held to the frame that presented measles as serious: back in the 1960s, health officials “didn’t think measles was funny, given that the virus could lead to pneumonia, encephalitis and even death,” one journalist wrote (Ross 2019). But individuals opposed to the politics of California’s new vaccine law rejected this version of collective memory, reinterpreting primary evidence from the past for themselves. “Remember when the entire Brady Bunch was wiped out by measles?!!” read one sarcastic meme (Ross 2019). Traditional news media had played a role in creating and sustaining collective memory of measles over a half-century during which, even as the disease’s past was subject to modifications and omissions, its severity was consistently amplified. But in this instance of measles reporting, the new media environment offered space for media producer-consumers to create and share the specific, partisan social memory that most aligned with their preferred vaccination ideology (Edy 2014).

Michael Schudson argued that monolithic and all-powerful versions of the past won’t triumph in pluralistic societies where conflicting views can emerge, find audience, and survive (1992). With measles, the Brady Bunch story and the trend it represents suggest a monolithic past may now be giving way. This possible shift follows five decades of reporting in which journalists firmly shaped public understanding of measles as a severe disease in urgent need of prevention via vaccination. Journalists did so using familiar and available frames, which supported a set of causal stories that shifted responsibility for measles’ spread from nature to the inadvertent party, government or individual, and then to the intentional culprit, the privileged, science-rejecting individual. Journalists also did so by communicating a past, constructed of decontextualized fragments, that supported the frames and causal stories transmitted through their reporting.

This presentist project was realized in part through a pattern that Edy has documented: as the proportion of the public able to check public memory against personal memory declines over time, journalistic authority over the past expands (2006: 105–106). This may be giving way, but as our analysis shows, for more than five decades of post-vaccine measles reporting, it held. This holds potential significance for journalistic coverage of other vaccine-preventable diseases, which move from autobiographical to collective memory at their own pace. The most recent disease to become vaccine preventable, Covid-19, was framed as such even before its vaccine was developed. As this disease shifts from its pre- to post-vaccine phase, journalistic reporting will likely have just as powerful and lasting effects on public understanding of its present—and its past.

## Endnotes

^1^All data were downloaded from ProQuest Historical Newspapers and NewsBank Access World News databases.

^2^After 2000, a large number of news reports about autism mentioned measles, in reference to the theory that the measles-containing vaccine then in use was a cause of autism, but as these articles were not about measles we omitted them from this analysis.
